# Notes and News

**Published:** 1893-04-15

**Authors:** 


### Notes and News.

Collected and Communicated.

A feature in the forthcoming festival of the North-Eastern Hospital for Children will be the presence of lady stewards. There is a committee of ladies in connection with the hospital, and such good work is done by this body, that we feel sure excellent results will accrue from obtaining further co-operation from the ladies.

We are glad to learn that the Hostel of St. Luke, the Nursing Home for the Clergy in connection with the Church House, of which the Bishop of London is President, Princess Christian Patroness, and Canon Cooper Secretary, is prospering. Patients have been admitted since the close of last year, and those who have made use of the benefits the Hostel offers testify in warm terms to their appreciation and gratitude. The Hostel is likely to become a real blessing to the poorer clergy especially, whilst the better off can be sure of all skilful attention and comfort also. We hope to hear that the proposed country Convalescent Home will be secured before very long.

Our readers will remember the introduction we gave them to "Leo," the canine collector for the Cork Children's Hospital. We have now to draw their attention to the excellent life work of another dog friend to charities. We regret that it is only after his demise, which occurred on the eve of intended decoration, that "Nip," who has collected for the Bristol medical charities, was brought to our notice. "Nip" has collected over forty pounds for these objects during his lifetime, and in his time-honoured years friends and admirers contributed to present him with a silver collar as an order of merit. Poor "Nip," however, did not live to wear the well-merited distinction. He was, like the well-known dog collector at Paddington Station, an Irish terrier. Passengers at Paddington Station view with pleasure this charitable member of the canine species, as he moves about with an air of responsibility and importance on his charitable mission. In "Nip" and his brother Irish terriers have certainly found worthy examples.

Most foreign countries have definite rules for patients' payments in hospitals according to means. The absence of any universal rule of the kind has caused much abuse of medical charity in England. In France special terms are made for well-to-do patients, and now a further regulation has been recommended by the Municipal Council of Paris, to the administrators of the hospitals, whereby an extra payment shall be made by patients undergoing operations.

The action whereby the Home Secretary has appointed two women as factory inspectors will meet the approval of intelligent and practical people. The habit of observing details is more common in women than men, and the training necessary for a thorough and comprehensive inspection is often lacking in the latter. If due care is exercised in the choice of women for the office of inspectors a great improvement should be speedily visible. Sensible and capable women will guard against the natural tendency of their sex to arbitrary interference when power is vested in them. A keen and fearless criticism, tempered by a due consideration of the circumstances of the case, is what is wanted, and what we believe will be forthcoming if judicious choice be made.

Dr. Brodie Sewell presided at a meeting held in the library of the *British Medical Journal* office on Monday to inaugurate a second Western Branch of the London and Counties Medical Protection Society. Dr. George B. Mead explained the principles upon which the society is founded, and gave a record of its rapid progress in the metropolis and the country. The society is now about a thousand strong, and its membership is increasing in all directions. The attendance of members of the profession was good, and a large number, who could not attend, expressed by letter their sympathy with the movement and their intention of becoming members. A long list of vice-presidents was proposed and carried, and Dr. Brodie Sewell was appointed president of the branch, with Dr. Sunderland, of Bruton-street, as secretary. The parent society of which Mr. Jonathan Hutchinson, F.R.S., is the president, and Dr. George Heron, F.R.C.P., honorary treasurer, is now securely established, and is rendering useful service to the profession in a variety of ways.

For the first time, in 1889, silk made from wood pulp was exhibited. It then received the highest award, and excited much interest. The experiment was just perfected, and time has been necessary for a larger development of the industry. The process was kept a secret by the inventor, M. de Chardonnet, who has built a mill at Besancon, in which some business men have joined. The process, which is a complicated one, is now disclosed, and the manufacture promises to prove a profitable one. The material used is wood pulp such as is used in the manufacture of certain kinds of paper. It is washed in acids and dried by alcohol. The pulp is further reduced to collodion. This collodion is ultimately forced through the needle point piercings of glass spouts, and issues in the form of a thread of wonderful fineness into a bath of ether and alcohol, which hardens it sufficiently. A thread as elastic and brilliant as silk is the result. The inflammable nature of the product is removed by passing it through a solution of ammonia. Whether this method of producing silk is less troublesome than the older one is doubtful, but the uncertainty of a crop will at any rate be obviated. The use of any particular kind of materia for clothing being a matter of fashion, great gains in this line of trade can never be safely reckoned on.

